# Moving beyond 90% Carbon Capture by Highly Selective Membrane Processes

**DOI:** 10.3390/membranes12040399

**Published:** 2022-04-01

**Authors:** Yang Han, W. S. Winston Ho

**Affiliations:** 1 William G. Lowrie Department of Chemical and Biomolecular Engineering, The Ohio State University, 151 West Woodruff Avenue, Columbus, OH 43210-1350, USA; han.779@osu.edu; 2 Department of Materials Science and Engineering, The Ohio State University, 2041 College Road, Columbus, OH 43210-1178, USA

**Keywords:** carbon capture, deep CCS, facilitated transport membrane, flue gas, membrane separation

## Abstract

A membrane-based system with a retentate recycle process in tandem with an enriching cascade was studied for >90% carbon capture from coal flue gas. A highly CO_2_-selective facilitated transport membrane (FTM) was utilized particularly to enhance the CO_2_ separation efficiency from the CO_2_-lean gases for a high capture degree. A techno-economic analysis showed that the retentate recycle process was advantageous for ≤90% capture owing to the reduced parasitic energy consumption and membrane area. At >90% capture, the enriching cascade outperformed the retentate recycle process since a higher feed-to-permeate pressure ratio could be applied. An overall 99% capture degree could be achieved by combining the two processes, which yielded a low capture cost of USD47.2/tonne, whereas that would be USD 42.0/tonne for 90% capture. This FTM-based approach for deep carbon capture and storage can direct air capture for the mitigation of carbon emissions in the energy sector.

## 1. Introduction

In the past two decades, methods to accelerate the decarbonization of the energy sector have been extensively investigated in order to limit the impact of global warming [1]. The removal of CO_2_ from fossil fuel combustion and the subsequent underground storage, commonly known as carbon capture and storage (CCS), is regarded as one of the most reliable and affordable options [2]. In this context, a target of “90% capture” has become ubiquitous not only in academic studies [3], but also in sustainability policies [4,5]. However, residual emissions still escape from the capture system, which need to be captured by “negative emissions” technologies such as direct air capture (DAC) [6] and bioenergy with carbon capture and storage (BECCS) [7]. Compared with carbon capture from large stationary sources, the negative emissions technologies often involve the separation or bioconversion of CO_2_ from air; nevertheless, the low CO_2_ concentration (ca. 410 ppm) requires energy-intensive separation systems with sizable footprints, which, in turn, exacerbate the energy sustainability [8,9,10].

Alternatively, a deep CCS concept with >90% capture has been proposed as a necessary pathway to decarbonize the power generations [11]. This scheme aims for a higher degree of CO_2_ removal so that the CO_2_ concentration in the exhaust flue gas approaches to that in air. For instance, coal flue gases typically contain ca. 13% CO_2_ [12,13], and residual emissions with 1–2% CO_2_ can slip through the capture system after 90% CO_2_ removal. If the capture is increased to 99%, the CO_2_ concentration in the resultant residual flue gas can be reduced to 0.1–0.2%. In order to achieve a carbon-neutral scenario, 99.7% of the CO_2_ must be captured. To distinguish from negative emissions technologies, methods capable of >90% capture from stationary sources are usually referred to as “near-zero emissions” technologies.

In the current literature archive, aqueous amine absorption is the only viable technology that can achieve a capture degree of up to 99.7% [14,15]. It has been shown that the capture cost for beyond 90% capture is only marginally higher than that at 90% capture, which makes this near-zero emissions technology competitive with DAC [16]. The research status is in contrast to the “partial capture” scenario, where membranes are widely investigated for 70–90% carbon capture [17,18,19,20,21]. In other words, membrane separation is ideal for bulk separation as it is a pressure-driven process [22,23]. When applied to removing dilute CO_2_, however, most polymeric membrane materials are limited by their insufficient CO_2_/N_2_ selectivities and thereby cannot achieve ≥95% CO_2_ purity. Consequently, complicated enriching cascade designs with repeated permeate recompressions are required [24].

Among all polymeric membrane materials, amine-containing facilitated transport membranes (FTMs) are potentially the best candidates to tackle the deep CCS. Firstly, the CO_2_ permeation is enhanced by the reversible reactions with amine carriers, but N_2_ is unable to react with the carriers and only permeates through the membrane based on the solution-diffusion mechanism [25,26]. The difference in reactivities results in greater CO_2_/N_2_ selectivities, which are usually 2–3 times higher than those of polymeric membranes without carriers [27,28,29,30,31]. Second, the CO_2_ transport performance in an FTM is dominated by the CO_2_–carrier reaction. The reaction rate typically increases with the reduction in the partial pressure of CO_2_ where the reacted carrier concentration is lowered and hence the free unreacted carrier concentration becomes abundant for the reaction, resulting in a higher CO_2_ permeance and CO_2_/N_2_ selectivity upon CO_2_ removal [32,33]. This so-called “mitigated carrier saturation phenomenon” has been observed in several FTM systems [32,34,35,36,37]. More importantly, the carrier structures can be tuned to enable a tolerance for carrier saturation up to 6–7 kPa CO_2_ partial pressure [32], which is attractive for >90% carbon capture (i.e., <1% CO_2_).

Previously, we have designed two membranes processes tailored for FTMs: (1) a retentate recycle process for 90% capture from coal flue gas [32]; and (2) a two-stage enriching cascade process to remove 90% CO_2_ from a dilute source with 1–2% CO_2_ [38]. Herein, we hypothesize that these two systems can be used in tandem to achieve an overall >90% capture degree where the retentate recycle process is responsible for the primary, bulk CO_2_ removal, while the enriching cascade is used to polish the residual CO_2_ as a secondary capture step. In order to assist the process design and techno-economic analysis, a benchmark FTM consisting of 15 wt.% poly(*N*-vinylforamide-*co*-vinylamine) and 85 wt.% 2-(1-piperazinyl)ethylamine sarcosinate is employed in this study as the membrane performance baseline [37,39]. The necessity of the combined systems is demonstrated by an analysis of the capture costs at 90–99% CO_2_ capture degrees. The marginal costs at >90% capture are also compared with DAC to justify the deep CCS approach.

## 2. Methods

### 2.1. Process Description

**Figure 1:**
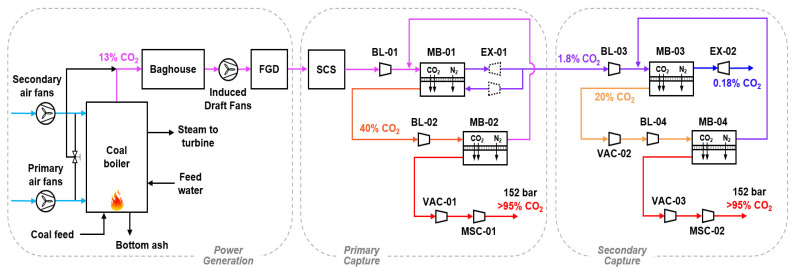
Schematic of the primary and secondary membrane systems for >90% CO_2_ capture. Initialisms: flue gas desulfurization (FGD), SO2 caustic scrubber (SCS), blower (BL), membrane (MB), expander (EX), vacuum pump (VAC), and multistage compressor (MSC).

Figure 1 shows the schematic of the primary and secondary membrane systems for >90% CO_2_ capture. The supercritical coal-fired power plant produces a flue gas containing 13.2% CO_2_ with particulate matter filtered by a baghouse collector and SO_2_ removed down to ca. 40 ppm by a flue gas desulfurization (FGD) unit. The SO_2_ is polished down to 3 ppm by an SO_2_ caustic scrubber (SCS) containing an aqueous solution of 20 wt.% NaOH. The flue gas is then first treated by the primary capture system aiming for 90% capture. The primary system features a two-stage process, with retentate recycle at the first stage, proposed in our previous work [32]. The flue gas is pressurized by blower BL-01 and enriched by the first membrane stage MB-01. After the energy recovery by the turboexpander EX-01, the CO_2_-depleted but N_2_-rich retentate is partially recycled to the permeate side as an internal sweep gas. The MB-01 permeate is repressurized by blower BL-02 and fed to the second membrane stage MB-02 with a permeate vacuum VAC-01, which further enriches the CO_2_ to ≥95% purity (dry basis). The retentate of MB-02 is recycled back to the feed side of MB-01.

The residual emissions from the primary capture system typically contain ca. 1.8% CO_2_. Should it be further decarbonized, a two-stage enriching cascade detailed in our previous publication [38] is used as the secondary capture system. In this case, the majority of MB-01 retentate is not expanded and is sent to the secondary capture system directly. Rather, only the portion used as the retentate recycle for MB-01 is expanded as shown in Figure 1. In the secondary capture system, another blower BL-03 further elevates the feed pressure for the enriching membrane stage MB-03. A vacuum VAC-02 is pulled on the permeate side in order to provide a higher transmembrane driving force. If the secondary system aims for 90% capture (i.e., 99% capture from the combined primary and secondary systems), the CO_2_ concentration in the MB-03 retentate can be reduced to ca. 0.18%, which is expanded by turboexpander EX-02. The permeate is compressed by blower BL-04 and further enriched to ≥95% purity by membrane stage MB-04. The MB-04 retentate is recycled back to the MB-03 feed.

**Figure 2:**
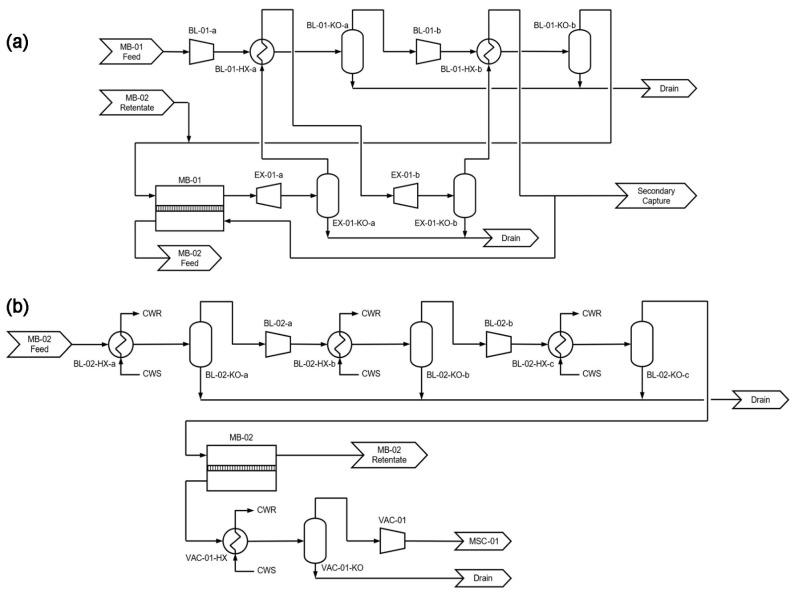
Detailed flow diagrams for the process equipment associated with (**a**) MB-01 for capture using the primary system only and (**b**) MB-02. Initialisms: cooling water supply (CWS), cooling water return (CWR), heat exchanger (EX), and knock-out vessel (KO).

Not shown in Figure 1 are the interstage cooling and heat integration of the blowers. One example is given by Figure 2, where the designs of the rotating equipment associated with MB-01 and MB-02 are detailed for capture using the primary system only. As shown, both BL-01 and EX-01 are split into two stages with interstage cooling and heating, respectively, in order to avoid excessive gas heating and to reduce the energy consumption. The expanded MB-01 retentate is used for the interstage cooling in BL-01, which reduces the cooling water demand of the capture system. Although BL-02 must involve water cooling, the heat duty is much less than that of BL-01 due to the much lower gas flow rate. A similar design principle is applied to the combined systems in tandem. Recall that EX-01 only expands the recycled retentate, and the remaining high-pressure retentate exiting MB-01 is mildly compressed by BL-03 and eventually expanded by EX-02. The expanded retentate in EX-02 is heat exchanged with BL-01 and BL-03 subsequently. Water cooling is used for BL-04 similar to that of BL-02.

### 2.2. FTM Modeling

It is known that the performance of an FTM depends on the CO_2_ partial pressure. Often, the CO_2_ permeance and CO_2_/N_2_ selectivity increases with the reduction in the partial pressure of CO_2_ due to the mitigated carrier saturation [32,33,37]. A homogeneous reactive diffusion model is used to describe the composition-dependent CO_2_ permeation [32]:

(1) $$P_{{\mathrm{CO}}_{2}}=P_{{\mathrm{CO}}_{2}}^{0}\left[1+\eta_{{\mathrm{CO}}_{2}}\left(\sqrt{1+\frac{p_{{\mathrm{CO}}_{2}}^{*}}{p_{{\mathrm{CO}}_{2}}^{h}}}-1\right)\right]$$

where $P_{{\mathrm{CO}}_{2}}$ is the CO_2_ permeance in a unit of GPU (1 GPU = 10^–6^ cm^3^ (STP) cm^–2^ s^–1^ cmHg^–1^), $P_{{\mathrm{CO}}_{2}}^{0}$ is the permeance at full carrier saturation, $\eta_{{\mathrm{CO}}_{2}}$ is the effective factor of facilitated transport, $p_{{\mathrm{CO}}_{2}}^{*}$ is the onset carrier saturation partial pressure, and $p_{{\mathrm{CO}}_{2}}^{h}$ is the CO_2_ partial pressure on the feed side. The N_2_ permeance ($P_{N_{2}}$) is assumed as a constant; therefore, the ideal CO_2_/N_2_ selectivity at full carrier saturation is defined as $\alpha^{0}$ = $P_{{\mathrm{CO}}_{2}}^{0}/P_{N_{2}}$.

Equation (1) implies that the local feed CO_2_ partial pressure dictates the CO_2_ permeance. Therefore, the CO_2_ permeance must be treated as a variable in the module modeling. Such a treatment has been detailed by the countercurrent and crossflow models developed in our previous work [32,40]. In this study, the countercurrent model was used for MB-01 while the crossflow model was employed for MB-02, MB-03, and MB-04.

### 2.3. Process Modeling

The operating conditions for the power plant, the primary and secondary membrane capture systems, and the benchmark FTM are listed in Table 1. Case B5A in the *Cost and Performance Baseline for Fossil Energy Plants Volume 1: Bituminous Coal and Natural Gas to Electricity (Revision 4, 2019)* [5] was used as the reference power plant with a net power of 650 MW_e_. The facilitated transport characteristics of the benchmark FTM were obtained by fitting the experimental data [38] using Equation (1), which will be further discussed in Section 3.1. For conciseness, the readers are referred to our previous publications for the detailed equipment schedules of the retentate recycle process [32] and the enriching cascade process [38]. All process simulations were performed using a MATLAB code developed in house with the Soave–Redlich–Kwong equation as the thermodynamic model for process streams [41,42]. Unless otherwise noted, the conditions summarized in Table 1 were used as the default.

### 2.4. Costing Modeling

Case B5B in the *Performance Baseline* [5] was followed for the cost modeling. The detailed costing procedures have been reported in our previous publications [32,38]. All costs are reported in 2018 US. Dollars (USD). The key assumptions are as follows:

1. An installed membrane skid cost of USD 44.6/m^2^ membrane area was assigned, including USD 21.5/m^2^ membrane element cost, USD 5.4/m^2^ housing cost, and 17.7/m^2^ installation cost, based on commercial-scale reverse osmosis plants [44];
2. A membrane lifetime of 4 years was assumed with a membrane replacement cost of USD 5.4/m^2^/yr;
3. A capital charge factor of 0.125 was applied to calculate the capital cost [5].

## 3. Results and Discussion

### 3.1. Performance of the Benchmark FTM

**Figure 3:**
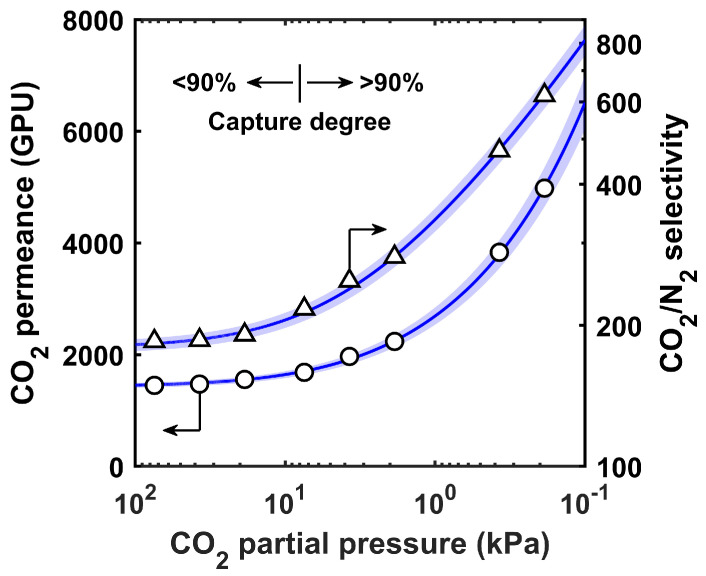
Increasing CO_2_ permeance and CO_2_/N_2_ selectivity with a decreasing partial pressure of the CO_2_ of the benchmark FTM as reported by Han and Ho [38]. The best fits and uncertainties based on Equation (1) are shown as solid lines and blue shades, respectively.

The mitigated carrier saturation of the benchmark FTM is illustrated in Figure 3. The CO_2_ permeances and CO_2_/N_2_ selectivities at different feed CO_2_ partial pressure values were reported by Han and Ho [38]. Equation (1) was used to fit the experimental data, and the fitting parameters, as listed in Table 1, were used for the process simulations. The benchmark FTM exhibited clear uprising trends of CO_2_ permeance and CO_2_/N_2_ selectivity with reductions in CO_2_ partial pressure. In other words, the CO_2_ separation became more selective upon the CO_2_ removal in the membrane module. An onset saturation pressure ($p_{{\mathrm{CO}}_{2}}^{*}$) of 7.5 kPa was obtained. This value was close to the CO_2_ partial pressure in the residual flue gas from the primary capture system (90% capture and 354.6 kPa (3.5 atm) feed pressure as in Table 1). Consequently, the secondary capture system could be considerably more selective than the primary one, which was well-suited to treat the dilute CO_2_ gas. For instance, with a partial pressure reduction from 38 to 7.5 kPa, the permeance increased from 1473 to 1684 GPU and the selectivity increased from 186 to 217. At a further reduced partial pressure of 0.4 kPa, the FTM showed an even greater permeance of 3832 GPU with a high selectivity of 472.

### 3.2. Capture Using the Primary System Only

#### 3.2.1. Effect of Retentate Recycle

In principle, the two-stage enriching cascade (see Figure 1) could be used for the primary carbon capture (i.e., removing 90% CO_2_ from the flue gas) [45,46]. Our recent work has also shown that it can achieve an overall 99% capture degree. Therefore, the necessity of the combined primary and secondary capture systems pivots on whether the retentate recycle process is more cost-effective for ca. 90% carbon capture. To this end, the CO_2_/N_2_ separation performance of the retentate recycle process was studied for 85–95% capture degrees. As one of the most important operating parameters, the percentage of the retentate recycle ($X_{r}$) varied between 0 and20%. At $X_{r}$ = 0, the retentate recycle process reduced to the two-stage enriching cascade. Based on the process optimization conducted in our previous work [32,38], a feed pressure ($p^{h}$) of 354.6 kPa (3.5 atm) was used for both MB-01 and MB-02, while a permeate pressure ($p^{l}$) of 81.0 kPa (0.8 atm) was applied to MB-02. All results presented in this section had a CO_2_ purity ≥95% through the primary capture system.

**Figure 4:**
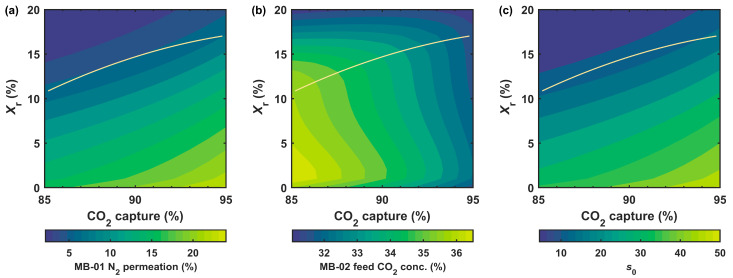
Effect of CO_2_ capture degree and retentate recycle ($X_{r}$) on (**a**) MB-01 N_2_ permeation, (**b**) feed CO_2_ concentration of MB-02, and (**c**) MB-01 dimensionless area ($s_{0}$ ) for the primary capture system. The solid lines are the optimal $X_{r}$ values for different capture degrees.

Figure 4 shows the percentage of N_2_ permeated through MB-01, the CO_2_ concentration of MB-02 feed, and the normalized membrane area ($s_{0}=A_{0}/\left(\frac{{\left.n^{h}\right|}_{A=0}}{p^{h}P_{{\mathrm{CO}}_{2}}^{0}}\right)$ [32]) of MB-01, where $A_{0}$ is the membrane area and ${\left.n^{h}\right|}_{A=0}$ the feed molar flow rate. The N_2_ permeation, or N_2_ loss, is defined as the percentage of N_2_ in the flue gas that permeates through MB-01 to the permeate side instead of through the retentate recycle. This value, in part, reflects the parasitic energy consumption of the retentate recycle process, or how much the compression energy of BL-01 can be recovered by EX-01. The flue gas (mainly N_2_) is pressurized to 354.6 kPa (3.5 atm) through the work of BL-01. With the thermal expansion in EX-01, the thermodynamic availability (i.e., work potential) carried by the N_2_ in the retentate can be recovered. However, the portion of the N_2_ that permeates through the membrane to the low-pressure side cannot be utilized for energy recovery. Consequently, the parasitic energy is adversely related to the N_2_ loss. As shown in Figure 4a, at a given capture degree, the N_2_ loss reduced considerably with increasing $X_{r}$. Therefore, the retentate recycle process (i.e., $X_{r}>0$) is advantageous over the enriching cascade (i.e., $X_{r}=0$) in terms of energy efficiency for treating the flue gas. For a fixed $X_{r}$, however, a higher N_2_ loss was observed at a higher capture. Therefore, the optimal $X_{r}$ value (based on the minimized capture cost) increased with the increasing capture as depicted by the solid lines in Figure 4.

It is worth noting that a proper amount of retentate recycle retards the N_2_ permeation but does not dilute the CO_2_ fed to MB-02. As seen in Figure 4b, the retentate recycle slightly increased the MB-2 feed CO_2_ concentration vs. the case with $X_{r}=0$, and it remained undiluted until the $X_{r}$ value was much higher than the optimal. Therefore, the separation performance of MB-02 was not adversely affected by the retentate recycle. Another feature exemplified in Figure 4b is that the optimal $X_{r}$ curve coincided with the second inflection points of the concentration isolines on the capture-$X_{r}$ plane. Therefore, the optimized system should possess the lowest possible N_2_ loss but not at the expense of the dilution of the MB-02 feed.

Although a proper retentate recycle did not change the CO_2_ concentration at the permeate outlet of MB-01, the internally recycled N_2_-rich effectively altered the permeate side flow pattern from the crossflow to the countercurrent, which particularly lowered the permeate CO_2_ concentration near the sweep inlet. Consequently, the retentate recycle drastically reduced the MB-01 membrane area as shown in Figure 4c. For instance, at 90% capture, the $s_{0}$ reduced by ca. three times after increasing $X_{r}$ from 0 to 15%.

#### 3.2.2. Costs at Different Capture Degrees

**Figure 5:**
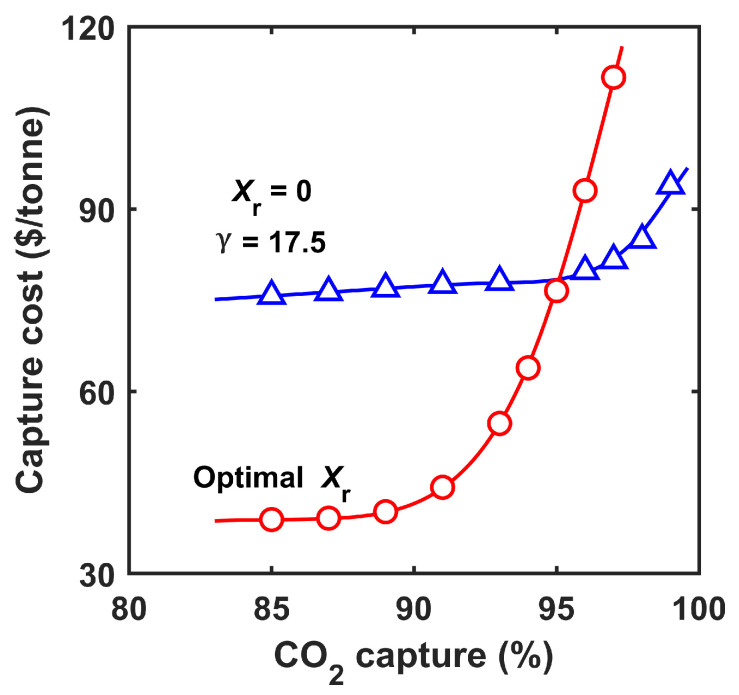
Capture costs of the primary system at different CO_2_ capture degrees with (1) $X_{r}$ = 0 (triangle symbols) and γ = 17.5 and (2) the optimal $X_{r}$ values (circle symbols), respectively.

The analysis in Figure 4 clearly shows that the retentate recycle process is superior to the enriching cascade for ca. 90% capture. However, the optimal $X_{r}$ curve tended to flatten out at a high CO_2_ capture degree. Increasing the capture from 90 to 95% led to ca. 22% more N_2_ loss and 7% less CO_2_ to MB-02 as feed, and more importantly, a 30% increase in the membrane area. The deteriorated energy efficiency and the more capital-intensive system resulted in a drastically increased capture cost beyond 90% capture. As illustrated in Figure 5, the capture cost increased from USD 42.0/tonne to USD 76.5/tonne when the capture degree was increased from 90 to 95%. At 97% capture, a prohibitively high capture cost of USD 111.6/tonne was observed even with retentate recycle.

The above results indicate that a higher transmembrane driving force is needed for a capture degree greater than 90%. Such an effect was demonstrated by setting $X_{r}=0$ and pulling a vacuum of 20.3 kPa (0.2 atm) on the permeate side of MB-01. Effectively, this system is equivalent to an enriching cascade with a feed-to-permeate pressure ratio ($\gamma =p^{h}/p^{l}$) of 17.5. The capture costs at different CO_2_ capture degrees were calculated for this system and the results are also shown in Figure 5. As expected, this system was not as competitive as the retentate recycle process at a lower capture degree of <95%. However, its capture cost did not increase as drastically on the high capture end, resulting in lower capture costs for >95% capture. For instance, a capture cost of USD 81.6/tonne was attained at 97% capture, and a further increase to 99% capture only led to a capture cost of USD 93.8/tonne. Apparently, the transmembrane driving force should be optimized based on the range of capture degrees. This observation motivated the use of the combined primary and secondary capture systems in tandem.

### 3.3. Capture Using Combined Systems in Tandem

#### 3.3.1. Separation Performance of the Secondary System

The separation performance of the secondary system was studied by assuming that 90% of the CO_2_ from the flue gas had been removed by the primary capture system with $X_{r}$ = 15% (see Table 1). In order to analyze the separation performance of the secondary system for treating the residual flue gas (1.85% CO_2_), the permeate CO_2_ purity and the dimensionless area of MB-03 were calculated for capture degrees of 50–90% and γ values of 10–25. The γ value was adjusted by varying the $p^{h}$ between 202.6 and 506.5 kPa (2 and 5 atm) while maintaining the $p^{l}$ at 20.3 kPa (0.2 atm). Combined with the 90% capture from the primary system, the overall capture, therefore, was 95–99%.

**Figure 6:**
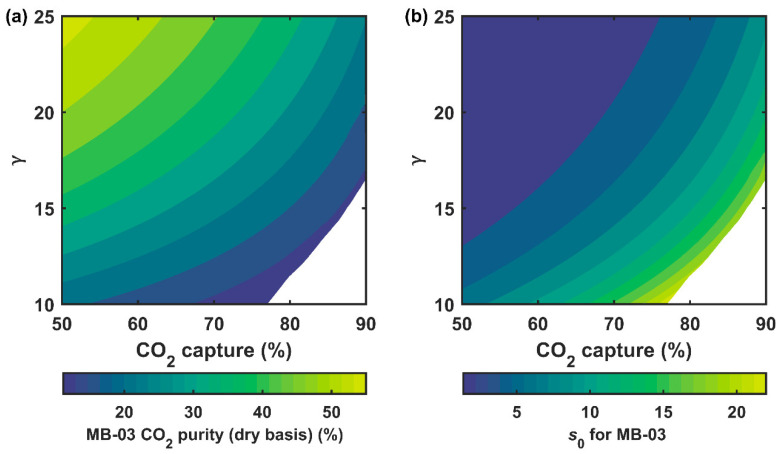
Effects of CO_2_ capture degree and feed-to-permeate pressure ratio (γ) on (**a**) the permeate CO_2_ purity (dry basis) and (**b**) dimensionless area ($s_{0}$ ) of MB-03 in the secondary capture system. The uncolored represents the regions where ≥95% purity in the final CO_2_ product is unattainable.

Figure 6a shows the calculated CO_2_ purities (dry basis) in the MB-03 permeate; the uncolored region represents the cases where ≥95% purity in the final CO_2_ product was unattainable. At a given capture degree, the MB-03 purity increased with increasing γ. In order to achieve 90% capture in the secondary system, the minimum γ required was ca. 16.5. In comparison, MB-01 in the primary system only employed a γ of 3.5, which further emphasized the different compression requirements of the two systems. In general, the residual flue gas could be enriched by MB-03 up to 40–50% at 50% capture, and the CO_2_ purity was at best ca. 20% at 90% capture. Nevertheless, the ≥20% CO_2_ stream fed as the feed to MB-04 yielded a permeate with >95% CO_2_. The limited driving force at high capture also affected the membrane area of MB-03. As shown in Figure 6b, a drastic increase in $s_{0}$ (i.e., denser, lighter isolines) was observed when the capture approached 90%. Even at γ = 25, increasing the capture from 50 to 90% led to a 7-time increase in the membrane area.

#### 3.3.2. Process Economics

The results in Figure 6 are consistent with our previous study on the enriching cascade [38], where a γ ≥ 22.5 (i.e., $p^{h}$ ≥ 4.5) is required for MB-03, especially at 90% capture. Despite the stringent operating conditions, the compression requirement of BL-03 could be significantly lower than that of BL-01 since the residual flue gas (i.e., the MB-01 retentate that is not recycled) was not expanded and thus delivered to the secondary system at 354.6 kPa (3.5 atm). Therefore, the compression ratio of BL-03 was reduced to 1.28, which could be achieved by a single-stage compression. In this regard, it is more advantageous for the secondary membrane system to be used in tandem with a membrane-based, pressure-driven primary system than other temperature-swing technologies such as solvents or sorbents.

**Figure 7:**
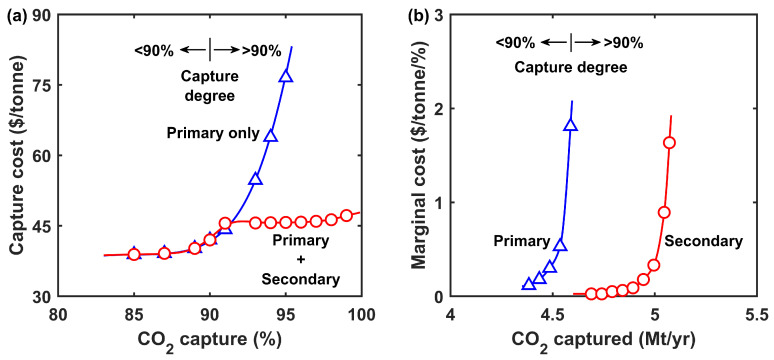
(**a**) Capture costs at different CO2 capture degrees for the primary system only and the combined primary and secondary systems in tandem. (**b**) Marginal costs of the combined systems.

The process economics of the combined process with the primary and secondary systems in tandem are shown in comparison with the primary system only in Figure 7a. The primary system with the retentate recycle process was used alone mainly for ≤90% capture, while the combined process was used for >90% capture. As seen, the combined process only led to a less pronounced cost increase from USD 42.0/tonne at 90% capture to USD 47.2/tonne at 99% capture. The 12% cost increase was significantly lower than using the primary system only throughout this range of capture, which makes the combined process attractive for deep CCS.

It is worth noting that it was difficult to clearly discern the relative contributions of the primary and secondary systems to the capture cost because of the relocation of EX-01 and the shared use of EX-02. Instead of a guesstimate approach, we calculated the marginal cost in a similar fashion as Brandl et al. [14]:

(2) $$\mathrm{Marginal}\;\mathrm{cost}=\frac{\partial C}{\partial \theta }\approx \frac{{\left.C\right|}_{\theta_{2}}-{\left.C\right|}_{\theta_{1}}}{\theta_{2}-\theta_{1}}$$

where C and θ are the capture cost and overall capture degree, respectively. Figure 7b shows the marginal costs of the combined systems plotted against the CO_2_ captured annually from the reference power plant as shown in Table 1. As seen, two distinct branches of marginal costs were observed with a crossover around 90% capture. Therefore, we assigned the left branch (blue color) to the primary system and the right one (red color) to the secondary system. At 90% capture, the marginal cost of the primary system was already as high as USD 1.8/tonne/%. Switching to the secondary system for the residual flue gas effectively reduced the marginal cost to <USD 0.1/tonne/%, which accounted for the significantly reduced overall capture cost. Even at 99% capture, the marginal cost was only USD 1.6/tonne/%. In comparison, our previous study showed a higher marginal cost of USD 8.9/tonne/% at 99% capture by using the secondary system alone [38]. This difference mainly stems from the relaxed compression ratio of BL-03 while in tandem with the primary system. Figure 7b also illustrates the relative scales of CO_2_ captured by the primary and secondary systems. At 99% capture, the marginal costs incurred by the secondary system only accounted for ca. 10% of the total CO_2_ captured. Therefore, the capture cost was less sensitive to the marginal cost of the secondary system.

Lastly, the importance of beyond 90% capture was demonstrated by a comparison with DAC. The marginal cost analysis suggested only a slight increase in capture cost by increasing the capture from 90 to 95%. Even for the extreme case where the capture increased from 98 to 99%, an additional capture cost of USD 1.6/tonne was needed, which was considerably lower than the USD 150–200/tonne for DAC [8,47,48]. Consequently, the deep CCS scheme in large stationary sources is useful to DAC for mitigating the carbon emissions in the energy sector.

## 4. Conclusions

An FTM-based system with the retentate recycle process in tandem with the enriching cascade was studied for >90% carbon capture from coal flue gas. The main conclusions attained in this study are as follows:

1. The retentate recycle process was advantageous for ≤90% capture owing to the reduced parasitic energy consumption and membrane area. In comparison, the enriching cascade was inferior for the partial capture scenario;
2. At >90% capture, the enriching cascade outperformed the retentate recycle pro-cess since a higher feed-to-permeate pressure ratio could be applied;
3. The combined process with primary and secondary capture systems in tandem could achieve a low capture cost of USD 47.2/tonne at 99% capture. The FTM-based deep CCS approach complements DAC.

## Figures and Tables

**Table 1:** Key plant operating conditions.

Parameter	Value
Power plant net power	650 MW_e_ (supercritical coal-fired power plant) [5]
Power plant capacity factor	0.85 [5]
Flue gas flow rate	32.71 kmol/s
Flue gas composition	13.21% CO_2_, 65.21% N_2_, 17.25% H_2_O with balancing O_2_ at 57 °C and 101.3 kPa (1 atm)
Primary CO_2_ capture spec	90% CO_2_ recovery, ≥95% CO_2_ purity
Residual flue gas flow rate	24.65 kmol/s
Residual flue gas composition	1.85% CO_2_, 85.70% N_2_, 7.72% H_2_O ^†^ with balancing O_2_
Secondary CO_2_ capture spec	90% CO_2_ recovery, ≥95% CO_2_ purity
Membrane temperature	67 °C
Feed pressure ^‡^	354.6 kPa (3.5 atm) for both MB-01 and MB-02; 456.0 kPa (4.5 atm) for both MB-03 and MB-04
Feed water content ^‡^	100% relative humidity at given feed temperature and pressure
Percentage of retentate recycle	15%
Vacuum pressure ^‡^	81.0 kPa (0.8 atm) for MB-02; 20.3 kPa (0.2 atm) for both MB-03 and MB-04
Heat transfer coefficient^‡^	60 W m^–2^ K^–1^ for BL-01 and BL-03; 100 W m^–2^ K^–1^ for BL-02 and BL-04 *
$P_{{\mathrm{CO}}_{2}}^{0}$	1431 GPU
$\alpha^{0}$	183
$\eta_{{\mathrm{CO}}_{2}}$	0.46
$p_{{\mathrm{CO}}_{2}}^{*}$	7.5 kPa

^†^ The residual flue gas is fully saturated with water vapor at 67 °C and 354.6 kPa (3.5 atm). It is then compressed by BL-03 and a minor amount of water is knocked out during the compression. ^‡^ Default operating conditions. * A lower heat transfer coefficient is assigned when the expanded retentate gas is used as the coolant in comparison with cooling water [43].

## Data Availability

The data presented in this study are available on request from the corresponding author.
